# Sexual risk taking among patients on antiretroviral therapy in an urban informal settlement in Kenya: a cross-sectional survey

**DOI:** 10.1186/1758-2652-14-20

**Published:** 2011-04-18

**Authors:** Anders Ragnarsson, Anna Mia Ekström, Jane Carter, Festus Ilako, Abigail Lukhwaro, Gaetano Marrone, Anna Thorson

**Affiliations:** 1 Karolinska Institutet, Department of Public Health Sciences, Division of Global Health (IHCAR), Stockholm, Sweden; 2 AMREF Kenya Country Programme, Nairobi, Kenya

## Abstract

**Background:**

Our intention was to analyze demographic and contextual factors associated with sexual risk taking among HIV-infected patients on antiretroviral treatment (ART) in Africa's largest informal urban settlement, Kibera in Nairobi, Kenya.

**Methods:**

We used a cross-sectional survey in a resource-poor, urban informal settlement in Nairobi; 515 consecutive adult patients on ART attending the African Medical and Research Foundation clinic in Kibera in Nairobi were included in the study. Interviewers used structured questionnaires covering socio-demographic characteristics, time on ART, number of sexual partners during the previous six months and consistency of condom use.

**Results:**

Twenty-eight percent of patients reported inconsistent condom use. Female patients were significantly more likely than men to report inconsistent condom use (aOR 3.03; 95% CI 1.60-5.72). Shorter time on ART was significantly associated with inconsistent condom use. Multiple sexual partners were more common among married men than among married women (adjusted OR 4.38; 95% CI 1.82-10.51).

**Conclusions:**

Inconsistent condom use was especially common among women and patients who had recently started ART, i.e., when the risk of HIV transmission is higher. Having multiple partners was quite common, especially among married men, with the potential of creating sexual networks and an increased risk of HIV transmission. ART needs to be accompanied by other preventive interventions to reduce the risk of new HIV infections among sero-discordant couples and to increase overall community effectiveness.

## Background

By December 2009, approximately 5.25 million people in low- and middle-income countries were receiving antiretroviral therapy (ART) - a 10-fold increase over five years [1]. However, many of the HIV and AIDS treatment programmes in low-income countries have not been coupled with efforts to support HIV prevention as it is not always a required approach [2].

The reduction in viral load in individuals treated with ART has led to optimistic expectations about the ability of treatment to limit the HIV epidemic, and several studies support ART as a prevention strategy [3]. However, this is still an ongoing international debate: several epidemiological models do not support this assumption [4,5]. In addition, several studies have reported that although genital shedding of HIV does decrease after initiation of ART, there is often incomplete suppression with a low correlation between HIV-RNA levels in blood compared with semen and vaginal fluids [6-8]. The risk of HIV transmission is also dependent on an individual's ability to adhere to the medical regimen, which affects both development of resistance to treatment drugs and viral load [9]. Additional crucial behavioural determinants of sexual transmission include inconsistent condom use, especially in combination with concurrent sexual partners [6-8,10-15].

Research on sexual behaviours of patients on ART shows contradictory results. Several studies from high-income countries, which predominantly focus on gay men, have shown increased risk taking with large numbers of high-risk sexual events taking place [16-21]. Recent systematic reviews did not show any association between ART initiation and increased sexual risk taking [22,23].

However, experiences from high-income settings are of limited value when addressing low-income, high-prevalence settings that are characterized by weak health systems, limited human resources capacity, and health services poorly adapted to large-scale ART delivery [24,25]. There are still relatively few studies undertaken in low-income settings, but among those published, there is an indication of many underlying contextual factors that hinder the individual from taking on sexual risk-reduction strategies [26,27]. The majority of ART patients in resource-poor settings are diagnosed at a very late stage of their HIV infection, implying high viral loads at the start of treatment [14,15,26,28]. As shown in another study recently undertaken in South Africa, almost half the participants just initiated on ART had unprotected sex at last intercourse [29].

This cross-sectional study was carried out among HIV-positive patients on ART in an urban informal settlement, Kibera in Nairobi, Kenya, a high-risk environment that has been given little attention, despite carrying a high HIV disease burden. The estimated overall HIV prevalence in Kenya is 7.8% [30], but in Kibera, it is estimated to be 12% [31]. As Africa is becoming more urbanized [32], places like Kibera provide important opportunities to better understand the HIV epidemic. Kibera has a high turnover of its inhabitants with resulting social coercion and high drop-out rates from ART programmes [9,26,33,34].

Research has also shown that people living in urban informal settlements, such as Kibera, have earlier sexual debuts, have more sexual partners, are more likely to use alcohol, and are less likely to adopt preventive measures against contracting HIV compared with urban residents in formal settings [35]. The aim of this study was to determine factors associated with sexual risk taking among people on ART in one of Africa's largest resource-poor, urban informal settlements (Kibera).

## Methods

### Study setting

Kibera in Nairobi is one of the largest informal settlements in Africa, comprising a young, multi-ethnic and mobile population of between 500,000 and 1,000,000 as a result of rapid urbanization (estimates of the population vary widely and no accurate data is available). The extremely high population turnover has had a profound impact in terms of reduced social cohesion. Kibera is a permanent fixture of mostly informal dwellings, where people live under deprived conditions with very limited access to basic services, such as education, healthcare and sanitation.

### Study population and inclusion criteria

The study was conducted among HIV patients attending a community-based health clinic in Kibera run by the African Medical and Research Foundation (AMREF). The clinic provides free treatment and care for people living with HIV and who are residents in Kibera. The study period started in September 2007 and ended in April 2009. All male and female patients above 18 years of age were eligible to participate in the study and were recruited consecutively at the AMREF clinic during their visits for treatment follow uA total of 515 patients (348 women and 167 men) consented to participate and provided complete data. None of the patients declined participation in the study.

### Data collection

A trained female research assistant administered structured questionnaires and undertook the interviews in Kiswahili at the AMREF clinic in Kibera. Each interview took approximately 20 to 30 minutes to conduct, and the patients were not reimbursed. The questionnaire was translated into Kiswahili and translated back into English several times to ensure correctness of content. Questions covered socio-demographic characteristic of the patients, such as tribe, age, sex, religion, time on ART, residential information and family structures.

Independent factors of relevance to the outcomes were explored by including questions on drug and alcohol use and health status. However, patients did not report any drug or alcohol use and these variables were thus not included in the model. Outcome variables were assessed by questions on sexual risk events, including number of sexual partners during the previous six months and consistency of condom use.

### Statistical analysis

SPSS for Windows (version 17.0) was used for statistical analysis. Data were routinely collected at the AMREF clinic by the research assistant, and entered consecutively into an SPSS data entry programme. Descriptive statistics were performed on socio-demographic characteristics and the outcome variables.

Sexual behaviour related outcomes were categorized and coded as follows: consistent condom use (yes="always", no="never or sometimes condom use"); and number of sexual partners in the previous six months (zero or one sexual partner in the previous six months vs. two or more sexual partners). Stochastic modelling has shown that for a fixed mean number of partners per individual, the distribution of the contact patterns, ranging from serial monogamy to concurrency, has a major influence on the speed of the spread of an epidemic [7,36]; therefore, we have chosen to dichotomize the number of sexual partners into these groups.

Mean and standard deviations were computed for numerical variables and proportions for categorical variables. Following the descriptive analysis, we performed bivariate and multiple logistic regression models to assess the association between explanatory variables and the outcomes of consistent condom use and a dichotomized number of sexual partners in the previous six months. The explanatory variables included in the bivariate analysis were: sex; age groups ("18-30", "31-40", "41-50", "51-70"); education ("never been to school", "primary school", "secondary school or more"); employment ("unemployed", "employed", "casual labour"); marital status ("married", "unmarried"); income per month ("less than 5000 Kenya shillings", "more than 5000 Kenya shillings", "uncertain"); time on ART in months ("1-6", "7-12", "13-18", "19-24", ">24"); and disclosed HIV status to wife/husband/partner, friend or family member ("Yes", "No").

Independent variables significant in bivariate analysis (chi-square or Fisher exact test) with a p value of <0.20 were included in the model and removed using a forward stepwise method (Wald Test with a removal level of significance of p <0.1 was applied). Odds ratios (ORs) and their 95% confidence intervals (CIs) were also computed. A value of p <0.05 was considered statistically significant and tests of significance were two sided. Hosmer-Lemeshow tests were computed to test the final model's goodness of fit; its p values were not significant for the consistent condom use model or for the multiple partners model. (A finding of non-significance corresponds to the conclusion that the model adequately fits the data.) Furthermore, the model was tested for collinearity between the independent variables but showed no significant results.

### Ethical considerations

Ethical approval for the study was obtained from the Kenya Medical Research Institute Ethical Review Committee. A local Swahili-speaking research assistant provided information on the aims of the study and asked for verbal, as well as written, informed consent from all study participants.

## Results

### Patient characteristics

A total of 515 enrolled HIV-positive patients (348 women and 167 men) with a mean age of 37 years participated in the study (Table 1). A descriptive analysis of the sample population showed that tribal backgrounds were very diverse and representative of the ethnic diversity in Kibera. Most patients reported being Christian (91%) and had lived in the Kibera informal settlement for more than five years (71%). The majority of patients had known their HIV status for more than one year (73%) and had received ART for more than one year (53%).

**Table 1:** Socio-demographic and clinical characteristics of patients at the ART clinic in the Kibera informal settlement

Characteristics	N (515)	All (%)	Men (%)	Women (%)
Women	348	67.6		
Men	167	32.4		
Age (mean ± sd)		37.3 (±8.1)	40.1 (±7.9)	36.0 (±7.9)
Religion				
Christian	467	90.6	88.6	91.6
Muslim	18	3.5	3.0	3.7
Other	30	5.8	8.4	4.6
Time in Kibera				
0-2 years	35	9.5	5.4	11.7
2-5 years	72	19.5	18.6	20.0
More than 5 years	262	71.0	76.0	68.3
Income level below 10,000 KES**	341	91.1	86.1	95.6
Time since first testing positive				
0-6 months	67	13.0	9.6	14.7
7-12 months	73	14.2	21.6	10.7
1-2 years	122	23.7	26.3	22.5
More than 2 years	252	48.9	42.5	52.2
Time on ART				
1-6 months	134	27.0	22.8	29.0
7-12 months	99	20.0	25.9	17.1
13-18 months	44	8.9	10.5	8.1
19-24 months	55	11.1	13.0	10.2
2 years >	146	31.1	27.8	35.6
Disclosed HIV status	446	86.6	87.4	86.2
Sex partners in the past 6 months				
0 partners	193	37.5	24.6	43.7
1 partner	273	53.0	58.7	50.3
2 or more partners	49	9.5	16.8	6.0
Married	253	49.2	24.0	63.7
Employment status				
Unemployed	229	44.5	56.9	38.5
Employed	160	31.1		
Casual labour	126	24.1		
Educational status				
Primary school	262	50.9	41.9	55.2
Secondary school or more	217	42.1	55.1	35.9
No formal education	36	7.0	3.0	8.9
Age < 40	335	65.0	52.1	71.3
Consistent condom use*				
Yes	264	71.7	81.8	65.3
No	104	28.3	18.2	34.7

*A total of 147 (28.5%) did not answer the condom question: of those, 123 were women (35% of total number of women) and 24 were men (14% of total number of men). X^2 ^test's p value <0.0001.

**10,000 Kenya shillings (KES) are approximately equal to US$125

The educational levels of the patients were relatively high: half of the patients had completed primary school (51%), and many had finished secondary school or even been to college (42%). Many people were unemployed (45%), with an income level of below 10,000 Kenya shillings (approximately US$125) a month. Inconsistent condom use was reported by 28% of patients while relatively few reported having two or more sexual partners (9.5%) in the previous six months.

### Inconsistent condom use

Close to one-third of patients reported inconsistent condom use, which indicates high numbers of potentially unsafe sexual events. Multiple regression analyses showed that gender and time on ART were important predictors of inconsistent condom use, with a trend showing that shorter ART use was significantly associated with inconsistent condom use. Patients who had been on ART for more than 19 months had a significantly decreased odds of inconsistent condom use compared with those who had been on treatment for less than six months (19-24 months: aOR 0.33; 95% CI 0.12-0.88; and >2 years: aOR 0.48; 95% CI 0.25-0.92). Female ART patients were three times more likely to report inconsistent condom use than male patients on ART (aOR 2.98; 95% CI 1.58-5.62).

Additionally, employment of any kind was associated with a possible protective effect against inconsistent condom use. Patients defining themselves as casual labourers reported inconsistent condom use significantly less often than unemployed patients (aOR 0.46; 95% CI 0.24-0.90); employed patients also had a decreased odds of inconsistent use than unemployed patients (aOR 0.59; 95% CI 0.32-1.10), even if not significant. No significant interactions were found between the independent variables. Bivariate and multiple analyses results are presented in Table 2.

**Table 2:** Multiple logistic regression for inconsistent condom use

Characteristics	Crude OR	95% CI	p value	aOR	95% CI	p value
Women*	2.38	1.45-4.00	0.001	2.98	1.58-5.62	0.001
Men						
Age	0.98	0.96-1.01	0.277	1.00	0.97-1.04	0.867
Religion						
Christian						
Muslim	0.55	0.12-2.56	0.445			
Other	0.78	0.30-2.00	0.601			
Time in Kibera						
0-2 years						
2-5 years	0.23	0.08-0.63	0.004			
More than 5 years	0.34	0.14-0.79	0.012			
Income below 10,000 KES**	0.58	0.18-1.64	0.276			
Knowledge of HIV status						
0-6 months						
7-12 months	0.67	0.30-1.52	0.341			
1-2 years	0.36	0.17-0.78	0.009			
More than 2 years	0.49	0.25-0.96	0.037			
Time on ART						
1-6 months						
7-12 months	0.82	0.43-1.58	0.561	0.97	0.49-1.92	0.934
13-18 months	0.60	0.24-1.48	0.265	0.71	0.27-1.82	0.471
19-24 months	0.29	0.11-0.76	0.012	0.33	0.12-0.88	0.026
2 years >	0.49	0.26-0.92	0.025	0.48	0.25-0.92	0.026
Disclosed HIV status	1.02	0.47-2.20	0.966			
Sex partners in the past 6 months						
0/1 partners	1.18	0.61-2.28	0.622	1.51	0.72-3.14	0.275
2 or more partners						
Married	0.98	0.64-1.56	0.917			
Unemployed						
Employed	0.86	0.51-1.44	0.559	0.60	0.33-1.11	0.103
Casual labour	0.51	0.27-0.93	0.028	0.46	0.24-0.90	0.024
Educational status						
Primary school						
Secondary school or more	0.54	0.20-1.44	0.218	0.53	0.18-1.53	0.24
No formal education	0.40	0.15-1.08	0.071	0.38	0.13-1.12	0.08

*A total of 147 (28.5%) did not answer the condom question: of those, 123 were women (35% of total number of women) and 24 were men (14% of total number of men). X2 test's p value <0.0001.

The results have been adjusted for number of sexual partners, age group and educational level. These variables were included in the final model even if the bivariate analysis did not show any significant association with the outcome, since they hypothetically could still be associated with the outcome.

### Multiple sexual partners

Multiple sexual partners (Table 3) is a key risk factor for HIV transmission. Our results showed a borderline significant effect of the interaction between marital status and sex on the multiple sexual partners outcome among patients receiving ART (p value = 0.054). The output of a logistic model, when there are interactions, is slightly different to the interpretations of output in models without interaction.

**Table 3:** Multiple logistic regression for having more than one partner during the previous six months

Characteristics	Crude OR	95% CI	p value	aOR	95% CI	p value
Women						
Men	3.14	1.72-5.71	0.000	4.38	1.82-10.51	0.001
Age	1.03	0.99-1.07	0.126			
Religion						
Christian						
Muslim	0.00	0.000-	0.998			
Other	0.64	0.15-2.77	0.548			
Time in Kibera						
0-2 years						
2-5 years	0.55	0.15-1.93	0.347			
More than 5 years	0.63	0.23-1.78	0.385			
Income level below 10,000 KES**	3.64	0.49-8.85	0.004			
Knowledge of HIV status						
0-6 months						
7-12 months	0.58	0.19-1.72	0.323			
1-2 years	0.70	0.28-1.77	0.453			
More than 2 years	0.62	0.27-1.41	0.252			
Time on ART						
1-6 months						
7-12 months	0.89	0.38-2.08	0.790			
13-18 months	0.79	0.25-2.53	0.696			
19-24 months	0.79	0.27-2.30	0.670			
2 years>	0.72	0.33-1.57	0.402			
Disclosed HIV status	1.30	0.58-2.90	0.528			
Married						
Unmarried	0.44	0.23-0.81	0.009	1.15	0.45-2.93	0.770
Unemployed						
Employed	0.52	0.24-1.11	0.092			
Casual labour	0.90	0.44-1.82	0.765			
Educational status						
Primary school						
Secondary school or more	4.70	0.62-35.51	0.134			
No formal education	2.98	0.38-23.08	0.297			
Consistent condom use*						
Yes						
No	1.18	0.61-2.28	0.662			
Sex: marital status	0.48	0.11-2.05	0.320	0.18	0.03-1.02	0.054
Constant				0.06		<0.001

*A total of 147 (28.5%) did not answer the condom question: of those, 123 were women (35% of total number of women) and 24 were men (14% of total number of men). X2 test's p value <0.0001.

In Table 3, the value of the constant represents the odds of having more than one sexual partner for the reference group, married women. Married men hence had a significantly higher odds of having more than one sexual partner compared to married women, OR = 4.376 (p = 0.001). Among unmarried people, men had lower odds of having more than one sexual partner compared to women, 4.376*0.178 = 0.78, though this association was not significant. Unmarried women also had a slightly elevated odds of having more than one partner compared to married women (OR = 1.15).

While married men were significantly more likely to have more than one partner compared to married women, this trend did not prevail comparing unmarried men and women, with a significant OR for the former, 4.376, but a not significant OR for the latter, 0.78. Thus, 1.150*0.178 = 0.20 (p = 0.036, 95%CI = 0.046-0.903) is the OR (having more than one sexual partner) for unmarried people versus married people in the group of males, who are less likely to have had multiple partners in the previous six months than men who are married; we did not find a significant difference among unmarried men and women.

The tendency to engage in multiple partnerships was thus strongly associated with male gender and marital status among male patients. In the group of women, marital status did not significantly influence whether or not they engaged in multiple partnerships. Bi- and multiple analyses are presented in Table 3.

## Discussion

In this study we analyzed sexual risk taking among HIV patients on ART, and found a concerning level of inconsistent condom use among men and women. Furthermore, a higher proportion of married men reported multiple sexual partners during the previous six months compared with women and unmarried men. Gender was identified as an important determinant of both inconsistent condom use and multiple sexual partners, which has been shown in other studies [26,27].

Women in this study were significantly more likely than men to report inconsistent use of condoms (aOR 3.03), even when adjusted for the reported number of partners. Even though condoms are widely available, either free or at a minimal cost, patients, both men and women, are likely to face a range of barriers to condom use. These might be due to lack of individual decision-making power in intimate relations or could relate to social pressure to conceive a child.

Other studies [26,37-41] have shown that reproductive desires play an important role in societies, and HIV-positive women and men may experience the pressure to fulfil normative social expectations. This is supported by findings in a qualitative study targeting the same population, where strong collective and personal wishes for reproduction were coupled with negative associations with condom use, such as "condoms are dirty or are for prostitutes only" [26].

The low level of use of condoms has recently been shown in another study among partnered HIV people, where 50% to 70% reported unprotected sexual intercourse [42]. This issue needs to be addressed in ART programme design. Low condom use among specific groups can thus be due to several different reasons, such as financial barriers and limited access, as well as stigma that hinders specific groups from taking preventive measures against HIV.

More married men (aOR 4.38; 95% CI 1.82-10.51) than married women reported multiple sexual partners during the six months preceding the interviews. These men are at risk of exposing themselves and others to reinfection or infection with HIV; sexual risk-reduction strategies are not well integrated into their behaviour. Similar findings have been reported from studies on male sexuality, where men have been identified as more vulnerable to ill health due to the construction of a risk-taking masculine ideal [43,44].

In addition, the fact that almost 20% of the HIV-positive men and 35% of the HIV-positive women on ART did not consistently use condoms illuminates a real and threatening source of ongoing HIV transmission within an informal settlement where social vulnerability is already high. Programmes that target such high-risk behaviours among identified HIV-positive patients on treatment are urgently needed to minimize the risk of HIV transmission.

The other key variable significantly associated with sexual risk behaviour was the duration of time in the ART programme. Furthermore, inconsistent condom use was associated with shorter time on ART. This may be associated with the fact that the majority were diagnosed with HIV and were in need of ART at the same point, and hence needed time in the ART programme to adjust to the idea of living with HIV [26]. This suggests that once patients have had a chance to accept and adjust to being HIV positive, counselling targeting adherence and nutrition, as well as sexual risk behaviours and risk-reduction strategies, appears to have an effect on behaviour.

On the other hand, these results are especially worrisome given the natural course of the disease: viral loads are usually very high at treatment initiation, and then decrease over time. Patients who have recently started on ART are thus especially important in terms of risk of transmission, and the results indicate a strong need to focus more on this vulnerable grouMoreover, we cannot account for patients who have dropped out from the treatment programme, and hypothetically there might be an association between staying in the programme and adapting to preventive messages; these issues merit further research specifically focusing on programme drop outs.

The time factor in ART programmes was also found to be important in two recent studies: individuals who were about to initiate ART or were just starting medication reported more unsafe sexual practices compared with those who were more treatment experienced [32,45]. Furthermore, the differences we found between the sexes highlight a need for a better contextual understanding of gender dynamics in prevention strategies, and for better support mechanisms to meet the specific needs of men and women. The importance of preventive interventions in conjunction with ART to reinforce safe sexual practices among patients has been identified [32,46,47], but more research is needed to build an evidence base for programmatic and policy decisions [23].

This cross-sectional study included retrospective, self-reported information on sexual behaviour and events, which are inherently sensitive issues and therefore may be biased due to stigma or social desirability. However, research assistants were trained to create good relations in order to minimize bias and help facilitate patients in answering questions. The recall period was six months for the number of sexual partners, which might affect people's ability to accurately remember details of sexual events. The possibility that patients' memories of risk behaviours would differ by outcome status in this study is highly unlikely, in turn minimizing the risk of a systematic bias.

As many as 28% of participants did not answer the question on condom use. Given the stigma attached to risk behaviour in the programme setting, we believe the missing data diluted our findings of associations with sexual risk taking. We could not explore concurrency in relationships as the total number of reported sexual partners during the previous six months could involve both concurrent and serial relationships.

## Conclusions

We found considerable levels of inconsistent condom use among patients on ART in this resource-poor, urban slum setting, especially among women (35%). We also found a higher proportion of married men than women reporting multiple partners during the previous six months. Our study represents patients who have entered a relatively well-functioning ART programme with an inherent support structure focusing on patient education and information [9], and yet sexual risk taking was prominent, particularly among those who recently started on ART.

Preventative strategies in ART programmes have to work within complex socio-cultural systems, especially in relation to gender dynamics. Safer sex practices are often a collective concern, where sexual practices do not work in isolation, but in strong relation to norms in the society, forming powerful barriers to sexual risk-reduction strategies. Our study shows that gender-specific needs of the patient, as well as time on ART, must be taken into consideration in counselling situations and in the design of ART programmes to reflect the realities of people and their sexual lives.

The roll out of ART cannot serve as a single preventive intervention, but must be linked with other preventive strategies for increased community effectiveness. Thus, weak infrastructure and challenged health service delivery in informal settlements must be considered by policy makers and the donor community when developing future interventions to avoid the risk of negative effects, such as increased HIV transmission.

## Competing interests

The authors declare that they have no competing interests.

## Authors' contributions

AR, AME, AT, JC and FI were part of the study design. AL was responsible for data collection. GM, AR and AT were responsible for data analysis. AR drafted the first version of the manuscript. All authors read and approved the final manuscript.
